# Targeting ASC in NLRP3 inflammasome by caffeic acid phenethyl ester: a novel strategy to treat acute gout

**DOI:** 10.1038/srep38622

**Published:** 2016-12-09

**Authors:** Hye Eun Lee, Gabsik Yang, Nam Doo Kim, Seongkeun Jeong, Yunjin Jung, Jae Young Choi, Hyun Ho Park, Joo Young Lee

**Affiliations:** 1 BK21plus team, College of Pharmacy, The Catholic University of Korea, Bucheon, 14662, Republic of Korea; 2 Daegu-Gyeongbuk Medical Innovation Foundation, New Drug Development Center, Daegu, 41061 Korea; 3 College of Pharmacy, Pusan National University, Busan, 46241, Republic of Korea; 4 School of Chemistry and biochemistry and Graduate school of Biochemistry, Yeungnam University, Gyeongsan, 38541, Republic of Korea

## Abstract

Gouty arthritis is caused by the deposition of uric acid crystals, which induce the activation of NOD-like receptor family, pyrin domain containing 3(NLRP3) inflammasome. The NLRP3 inflammasome, composed of NLRP3, the adaptor protein ASC, and caspase-1, is closely linked to the pathogenesis of various metabolic diseases including gouty arthritis. We investigated whether an orally administrable inhibitor of NLRP3 inflammasome was effective for alleviating the pathological symptoms of gouty arthritis and what was the underlying mechanism. In primary mouse macrophages, caffeic acid phenethyl ester(CAPE) blocked caspase-1 activation and IL-1β production induced by MSU crystals, showing that CAPE suppresses NLRP3 inflammasome activation. In mouse gouty arthritis models, oral administration of CAPE suppressed MSU crystals-induced caspase-1 activation and IL-1β production in the air pouch exudates and the foot tissues, correlating with attenuation of inflammatory symptoms. CAPE directly associated with ASC as shown by SPR analysis and co-precipitation, resulting in blockade of NLRP3-ASC interaction induced by MSU crystals. Our findings provide a novel regulatory mechanism by which small molecules harness the activation of NLRP3 inflammasome by presenting ASC as a new target. Furthermore, the results suggest the preventive or therapeutic strategy for NLRP3-related inflammatory diseases such as gouty arthritis using orally available small molecules.

Gout is a common cause of inflammatory arthritis that causes red, tender, hot, swollen joints and is characterized by severe, intense pain and most commonly affects the metatarsal-phalangeal joint at the base of the big toe. Over the past two decades, the prevalence of the western diet has increased the incidence of gout 2-fold, particularly in elderly populations^1^. However, the clinical uses of present used drugs are somewhat limited; after oral administration of colchicine, over 80% of patients experienced abdominal pain prior to full clinical improvement. In addition, the adverse effects of non-steroidal anti-inflammatory drugs (NSAIDs) are more pronounced in the elderly^2^. Moreover, these drugs do not cure gout, merely providing temporary pain relief. Newly developed anti-interleukin (IL)-1 drugs, such as anakinra, canakinumab, and rilonacept, have been investigated for use in patents with gout^3^,^4^. Although current anti-IL-1 treatments appear to be highly effective against acute gouty arthritis attacks, they also have major limitations, such as their high cost, inconvenient treatment routes and regimens, and side effects. Therefore, it is critical to investigate the inflammatory mechanisms implicated in the pathogenesis of gouty arthritis, and to develop more effective agents for its treatment.

Gout is caused by the deposition of uric acid crystals in the articular and peri-articular tissues^5^. The disease incidence is directly correlated with serum urate levels^6^. Recently, the receptor that responds to uric acid crystals and generates inflammatory signals has been identified: NOD-like receptor family, pyrin domain containing 3(NLRP3)^7^. NLRP3 is a member of the Nod-like receptor family(NLR) and detects microbial invasion and endogenous danger signals, including uric acid crystals. In the presence of these signals, NLRP3 forms an inflammasome with an adaptor protein, apoptosis-associated speck-like protein containing a CARD(ASC), and pro-caspase-1. Pro-caspase-1 is cleaved to generate caspase-1, its active form, and caspase-1 cleaves pro-IL-1β precursor to generate active IL-1β, which is secreted into the extracellular environment. A previous study showed that macrophages from mice deficient in NLRP3 inflammasome components were unable to secrete active IL-1β following stimulation with uric acid crystals^7^. Articular inflammation induced by MSU crystals was dependent on NLRP3 inflammasome; in NLRP3-, ASC- or caspase-1-deficient mice, neutrophil influx was abrogated, and the production of gout-related cytokines was reduced^8^. Because IL-1β is the major effector cytokine produced in gout^3^ and because NLRP3 inflammasome activation is strongly implicated in the pathogenesis of gout^7^, repression of the NLRP3 inflammasome could provide an effective therapeutic strategy for gout.

This observation prompted us to search for available small-molecule inhibitors of the NLRP3 inflammasome that could be administered orally. We intended to find the compound to inhibit inflammasome activation among phytochemicals. We screened various anti-inflammatory phytochemicals and caffeic acid phenethyl ester (CAPE) was one of the most effective inhibitors of NLRP3 inflammasome. CAPE is an active component of honeybee propolis and is well known for its anti-inflammatory property^9^. Therefore, we investigated whether CAPE could suppress uric acid-induced activation of the NLRP3 inflammasome, using bone marrow-derived primary macrophages (BMDMs) and *in vivo* animal gout models. Our results would provide a novel preventive or therapeutic strategy using anti-inflammatory phytochemicals targeting NLRP3 inflammasome for the treatment of metabolic diseases such as acute gout.

## Results

### CAPE suppresses uric acid crystal-induced NLRP3 inflammasome activation in bone marrow-derived primary macrophages

**Figure 1:**
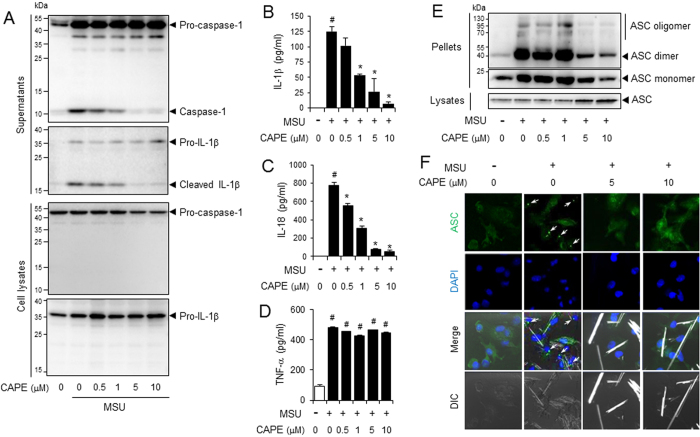
CAPE suppresses the MSU crystals-induced activation of the NLRP3 inflammasome in primary macrophages. Bone marrow-derived macrophages (BMDMs) were primed with LPS (500 ng/ml) for 4 hr. The cells were treated with CAPE for 1 hr and then stimulated with monosodium uric acid (MSU) crystals (500 μg/ml) for (**A**) 4.5 hr or (**B**–**E**) 6 hr. In (**A**), the cell culture supernatants and cell lysates were immunoblotted for pro-caspase-1, caspase-1 (p10), pro-IL-1β, and IL-1β. In (**B**, **C** and **D**) the cell culture supernatants were analyzed for secreted IL-1β, IL-18, and TNF-α using ELISA. The values represent the means ± SEM (n = 3). ^#^Significantly different from vehicle alone, p < 0.05. *Significantly different from MSU alone, p < 0.05. In (**E**), the cell lysates and crosslinked pellets were resolved using SDS-PAGE and were immunoblotted for ASC. In (**F**), the cells were fixed, permeabilized and stained for ASC (green), and the nuclei were stained with 4′,6-diamidino-2-phenylindole (DAPI; blue). The arrows indicate ASC speckles. The data are representative of three independent experiments. CAPE, caffeic acid phenethyl ester; MSU, monosodium uric acid crystals. DIC, differential interference contrast.

We first investigated whether CAPE could block the activation of NLRP3 inflammasome induced by uric acid crystals. BMDMs were first primed with LPS. To exclude the possibility that CAPE might affect LPS-mediated signaling pathways, CAPE was added after washing out the LPS. After pre-treatment with CAPE, cells were further stimulated with MSU crystals. CAPE alone or in combination with MSU did not reduce cell viability nor induce cell death in BMDMs (Supplemental Figure 1). CAPE inhibited MSU crystal-induced cleavage of pro-caspase-1 and pro-IL-1β, to caspase-1(p10) and IL-1β, respectively, in the cellular supernatants (Fig. 1A). The cleavages of pro-caspase-1 to caspase-1 and of pro-IL-1β to IL-1β are considered hallmarks of inflammasome activation. In addition, CAPE consistently reduced MSU crystals-induced secretion of IL-1β in a dose-dependent manner (Fig. 1B). Furthermore, CAPE suppressed MSU crystals-induced production of IL-18, another cytokine produced upon inflammasome activation (Fig. 1C). CAPE did not affect the mRNA levels of IL-1β and IL-18 in BMDMs stimulated with MSU, showing that the decrease of IL-1β and IL-18 by CAPE was not dependent on transcriptional regulation (Supplemental Figures 2A and 2B). CAPE did not affect the secretion of TNF-α, the release of which is independent on the inflammasome, in BMDMs stimulated by MSU (Fig. 1D).

ASC forms oligomers in response to NLRP3 activators^10^, as another measure of inflammasome activation. CAPE suppressed MSU crystals-induced formation of ASC oligomers in BMDMs (Fig. 1E). Confocal microscopy analysis consistently showed that CAPE reduced MSU crystal-induced formation of ASC speckles (Fig. 1F and Supplemental Figure 3). CAPE did not affect the mRNA levels of ASC, suggesting that CAPE did not inhibit the synthesis of ASC (Supplemental Figure 4).

We determined whether CAPE impacted the activation of NLRP3 inflammasome in human cells. CAPE suppressed the degradation of pro-caspases-1 to caspase-1(p10) and the cleavage of pro-IL-1β to IL-1β induced by MSU crystals in THP-1, human monocyte cell line (Supplemental Figure 5A). MSU crystals-induced IL-1β secretion was also decreased by CAPE in THP-1 cells (Supplemental Figure 5B). The results show that CAPE suppressed the activation of NLRP3 inflammasome in murine macrophages and human monocytic cells.

Uric acid crystals are phagocytosed and destabilize the phagosome, which activates the NLRP3 inflammasome^7^. However, other NLRP3 activators activate the NLRP3 inflammasome via different upstream pathways. For example, adenosine triphosphate (ATP), triggers NLRP3 inflammasome activation by binding to purinergic receptors such as P2X7, thereby increasing potassium efflux^11^. Nigericin, a microbial toxin derived from Streptomyces hygroscopicus, decreases intracellular potassium levels by acting as a potassium ionophore independent of receptor activation^12^. Therefore, we examined whether CAPE could inhibit NLRP3 inflammasome activation mediated by other activators, such as ATP and nigericin. CAPE suppressed ATP-induced cleavage of pro-caspase-1 and pro-IL-1β to caspase-1(p10) and IL-1β in BMDMs (Supplemental Figure 6A) and reduced ATP-induced secretion of IL-1β and IL-18 (Supplemental Figures 6B and 6C). In addition, CAPE suppressed nigericin-induced cleavage of pro-caspase-1 and pro-IL-1β and secretion of IL-1β and IL-18 in BMDMs (Supplemental Figures 6A, 6B, and 6C). These results show that CAPE suppresses NLRP3 inflammasome activation mediated by other activators, such as ATP and nigericin in macrophages.

### Oral administration of CAPE attenuates uric acid crystals-induced inflammasome activation in a mouse air pouch inflammation model

**Figure 2:**
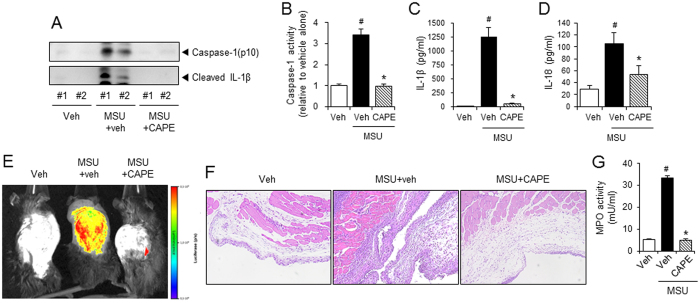
Oral administration of CAPE attenuates MSU crystals-induced NLRP3 inflammasome activation in a mouse air pouch model. (**A–E**) Air pouches were formed on the dorsa of C57BL/6 mice by injecting air twice. The mice were orally administered CAPE (30 mg/kg) or vehicle (Veh, 0.02% DMSO in water). After 1 hr, MSU crystals (3 mg/ml in PBS/mouse) or PBS alone were injected into the air pouches. After 6 hr, the pouch exudates were harvested and the supernatants were analyzed by (**A**) immunoblotting for caspase-1(p10) and IL-1β, (**B**) caspase-1 enzyme activity assay, and ELISAs for (**C**) IL-1β, and (**D**) IL-18. (**E**) Bone marrow-derived immortalized macrophages that had been transfected with the iGLuc luciferase reporter plasmid were injected into the air pouches. After 3 hr, the mice were orally administered CAPE (30 mg/kg) or vehicle. After 1 hr, MSU crystals (3 mg/ml in PBS/mouse) or PBS alone were injected into the air pouches. After 6 hr, luminescence derived from iGLuc-luciferase expression was assessed by *in vivo* imaging analysis using an Xtreme system (Bruker). (**F**) The air pouch tissue was fixed for histological examination using H&E staining. The purple dots represent infiltrated neutrophils. (**G**) Myeloperoxidase (MPO) activity, which reflects neutrophil recruitment, was assessed in the air pouch exudates. The values in the bar graphs represent the means ± SEM (n = 3–6 mice). ^#^Significantly different from vehicle alone, p < 0.05. *Significantly different from MSU alone, p < 0.05. Veh, vehicle.

We attempted to confirm the suppressive effects of CAPE on NLRP3 inflammasome *in vivo* using a mouse air pouch model. After air pouches were formed on the backs of the mice, the mice were orally administered 30 mg/kg of CAPE. One hour later, MSU crystals were injected into the air pouch to activate the NLRP3 inflammasome, resulting in the production of cleaved caspase-1 and IL-1β in the air pouch exudates (Fig. 2A). Oral administration of CAPE abolished MSU crystal-induced cleavage of pro-caspase-1 and pro-IL-1β in air pouch exudates (Fig. 2A). In addition, caspase-1′s enzymatic activity was reduced in air pouch exudates isolated from mice that had been administered CAPE (Fig. 2B). MSU-induced increases in IL-1β and IL-18 levels in air pouch exudates were also reduced by oral administration of CAPE (Fig. 2C and D).

Luminescence imaging-based *in vivo* scan analysis further supported these results. Bone marrow-derived immortalized macrophages transfected with the iGLuc luciferase reporter gene^13^, were injected into the air pouches. Injecting the air pouches with MSU crystals increased their luminescence, demonstrating iGLuc reporter gene activation (Fig. 2E). In contrast, oral administration of CAPE greatly reduced the luminescence generated by MSU crystals injection (Fig. 2E). These results provide *in vivo* evidence for the suppressive effects of CAPE on NLRP3 inflammasome induction by uric acid crystals.

CAPE diminished neutrophil infiltration in the air pouch tissue and exudates, as shown by histological examination and myeloperoxidase activity, respectively (Fig. 2F and G), demonstrating that CAPE treatment attenuates the inflammatory responses induced by uric acid crystals by suppressing NLRP3 inflammasome activation.

### Oral administration of CAPE prevents uric acid crystals-induced gout in mice by blocking NLRP3 inflammasome activation

**Figure 3:**
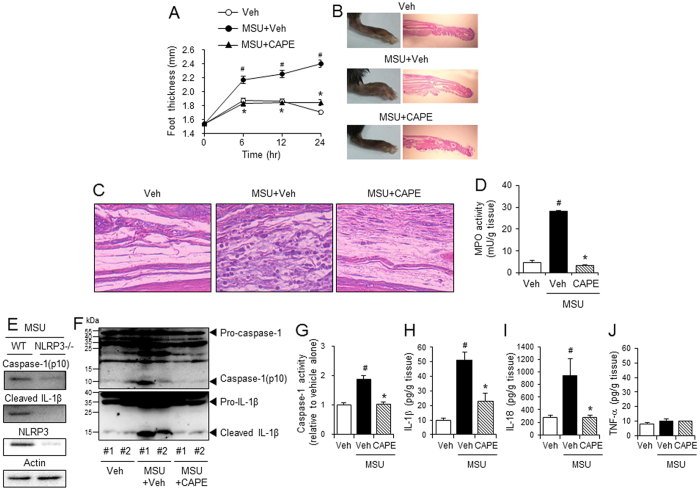
Oral administration of CAPE prevents MSU crystals-induced gout in mouse foot by blocking NLRP3 inflammasome activation. Mice were orally administered CAPE (30 mg/kg) or vehicle (Veh, 0.02% DMSO in water). After 1 hr, MSU crystals (2 mg/0.1 ml of PBS/mouse) or PBS alone were subcutaneously injected into the pad of the right hind foot of each mouse. After 24 hr, the footpad tissue was collected for analysis. (**A**) Time course of foot thickness. (**B**) Representative photographs and H&E staining of the hind feet. (**C**) Infiltrated neutrophils in the hind foot tissue appear as purple dots in H&E staining (400X). (**D**) Supernatants from the foot tissue lysates were analyzed for myeloperoxidase (MPO) activity. (**E**) MSU crystals (2 mg/0.1 ml of PBS/mouse) or PBS alone were subcutaneously injected into the pads of the right hind feet of wild-type (WT) and NLRP3-knockout mice. The foot tissue was analyzed by immunoblotting for caspase-1(p10), IL-1β, NLRP3, and actin. (**F–J**) The foot tissues from Fig. 3A were subjected to immunoblotting for pro-caspase-1, caspase-1(p10), pro-IL-1β, and IL-1β, a caspase-1 enzyme activity assay, and ELISAs for IL-1β, IL-18, and TNF-α. The values in the line and bar graphs represent the means ± SEM (n = 3 mice). ^#^Significantly different from vehicle alone, p < 0.05. *Significantly different from MSU alone, p < 0.05. Veh, vehicle.

Next, we investigated whether CAPE’s suppression of NLRP3 inflammasome could be applied to treat gout. A gout mouse model was generated by injecting MSU crystals into a mouse’s hind foot; the injection led to increased foot thickness and neutrophil infiltration into the foot tissue (Fig. 3A–D). Oral administration of CAPE reduced the foot thickness to normal levels (Fig. 3A and B). CAPE blocked MSU crystal-induced recruitment of neutrophils to foot tissues, as shown by histological examination of the foot tissue and by analysis of myeloperoxidase activity in foot tissue homogenates (Fig. 3C and D). These results show that oral administration of CAPE attenuates the inflammatory symptoms of gout caused by the injection of uric acid crystals in mice.

Injection of MSU crystals induced the degradation of pro-caspase-1 to caspase-1(p10) and the cleavage of pro-IL-1β to IL-1β in foot tissue homogenates of wild-type mice (Fig. 3E), but not in foot tissue of NLRP3 knockout mice (Fig. 3E). These results indicate that in the mouse foot, the MSU crystal-induced production of caspase-1(p10) and IL-1β depends on NLRP3 inflammasome activation. We examined whether CAPE could suppress uric acid crystals-induced NLRP3 inflammasome activation in gout. Oral administration of CAPE prevented the cleavage of pro-caspase-1 to caspase-1(p10) and of pro-IL-1β to IL-1β in foot tissue injected with MSU crystals (Fig. 3F). Caspase-1 enzyme activity in foot tissue homogenates consistently increased as a result of MSU crystal injection, but this increase was abolished by CAPE treatment (Fig. 3G). Furthermore, CAPE decreased MSU crystal-induced production of IL-1β and IL-18 in foot tissue homogenates (Fig. 3H and I). In contrast, neither MSU crystal injection nor CAPE treatment altered TNF-α levels (Fig. 3J), indicating that TNF-α may not play an important role in the inflammatory symptoms of uric acid crystal-induced gout.

Consistent with the results from the air pouch inflammation model, oral administration of CAPE effectively alleviated the inflammatory symptoms of uric acid crystal-induced gout model in mice. In addition, the suppressive effects of CAPE on gout were mediated by its blocking NLRP3 inflammasome activation in the mouse foot.

### CAPE directly binds to ASC

**Figure 4:**
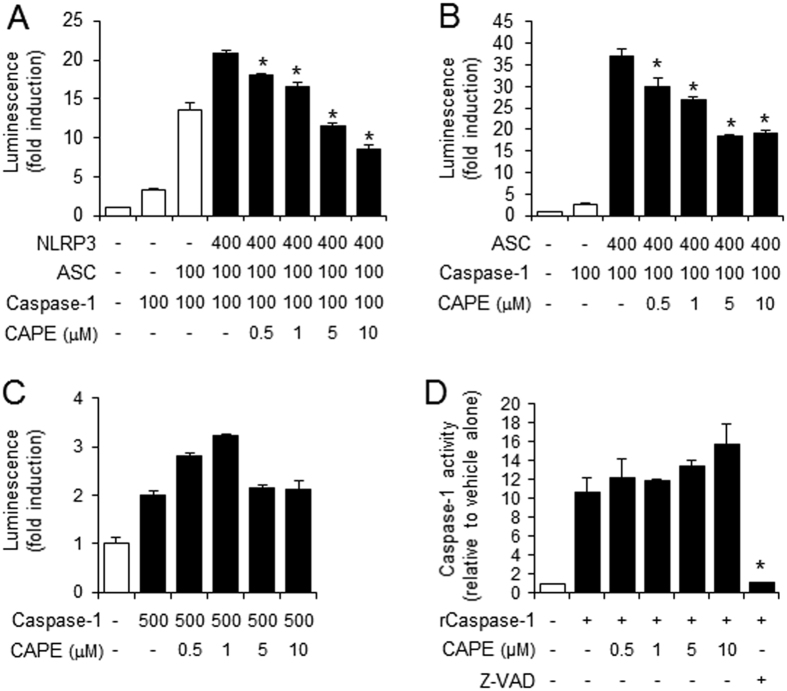
The suppression of inflammasome activation by CAPE is dependent on ASC. (**A–C**) 293T cells were transiently transfected with the iGLuc luciferase reporter plasmid (100 ng) and expression plasmids. Luminescence derived from iGLuc activation in each sample was normalized by β-galactosidase activity transfected as an internal control in each sample. (**A**) *Significantly different from NLRP3 + ASC + caspase-1, 0.5: p = 0.0064, 1–10: p =< 0.0001. (**B**) *Significantly different from ASC + caspase-1, 0.5: p = 0.0036, 1: p = 0.0001, 5–10: p =< 0.0001. (**D**) *In vitro* assay for caspase-1 enzyme activity was performed using a fluorometric caspase-1 assay kit with recombinant human caspase-1 (rCaspase-1; Bio-vision) in the presence or absence of CAPE or Z-VAD-FMK according to the manufacture’s instruction. The fluorescence was recorded at 400 nm after excitation at 505 nm with SpectraMaxM5 (Molecular Devices, Sunnyvale, CA). *Significantly different from rCaspase-1 alone, p = 0.0007. The values represent the means ± SEM (n = 3).

We investigated the mechanism by which CAPE suppresses NLRP3 inflammasome activation. To narrow down the inflammasome components that CAPE might target, we reconstituted the NLRP3 inflammasome complex in 293T cells by overexpressing each component, and the expression of iGLuc luciferase reporter gene was measured as an indicator of inflammasome components activation. When 293T cells were transfected with all three components, CAPE was able to suppress the expression of iGLuc luciferase reporter gene induced by NLRP3 plus ASC plus caspase-1 (Fig. 4A). When 293T cells were transfected with ASC and caspase-1 without NLRP3, CAPE could still block the expression of iGLuc luciferase (Fig. 4B). However, when 293T cells were transfected only with caspase-1, CAPE did not inhibit the expression of iGLuc luciferase (Fig. 4C), suggesting that CAPE does not target caspase-1. An *in vitro* caspase-1 enzyme activity assay confirmed that CAPE did not directly inhibit caspase-1 activity (Fig. 4D). Since the inhibitory effects of CAPE were observed when ASC was present, an inhibitable component would be narrowed down to ASC. These suggest that the target of CAPE may be ASC, not NLPR3 nor caspase-1.

**Figure 5:**
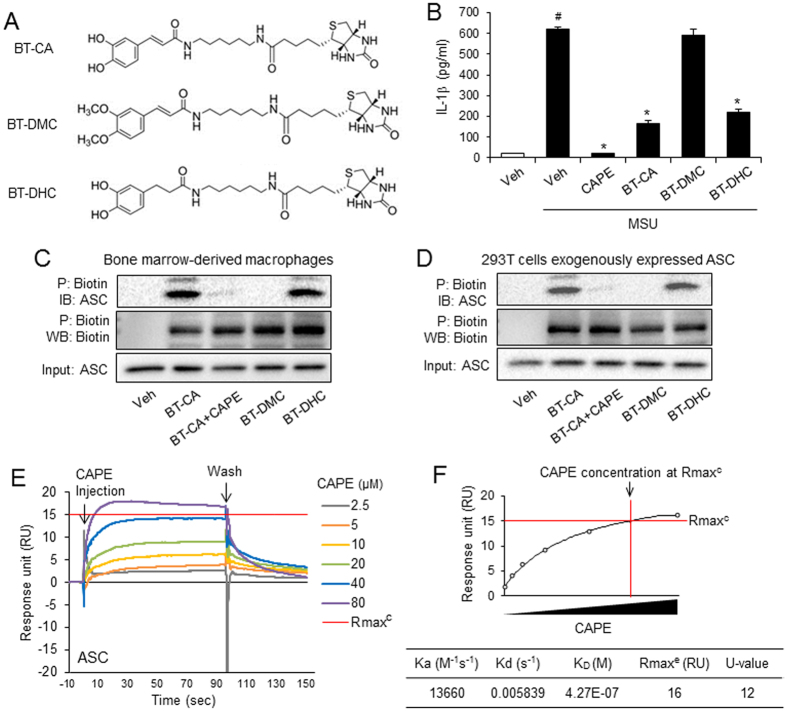
CAPE associated with ASC expressed endogenously and exogenously in the cell. (**A**) The structure of biotin-tagged CA (BT-CA), biotin-tagged DMC (BT-DMC), and biotin-tagged DHC (BT-DHC). (**B**) LPS-primed BMDMs were treated with CAPE, BT-CA, BT-DMC, and BT-DHC (10 μM) for 1 hr and then stimulated with monosodium uric acid (MSU) crystals (500 μg/ml) for 6 hr. The cell culture supernatants were analyzed for secreted IL-1β using ELISA. The values represent the means ± SEM (n = 3). ^#^Significantly different from vehicle alone, p < 0.0001. *Significantly different from MSU alone, p < 0.0001. (**C**) After BMDM cell lysates were treated with BT-CA, BT-DMC, and BT-DHC (1 μM) at room temperature for 4 hr, cell lysates were precipitated with NeutrAvidin beads and subjected to immunoblotting analysis. The amount of ASC expression in cell lysates were determined as “input”. CAPE (1 μM) was added to cell lysates treated with BT-CA. (**D**) After 293T cells were transfected with ASC-expression plasmids, the cell lysates were treated with BT-CA, BT-DMC, and BT-DHC (1 μM) at room temperature for 4 hr. The cell lysates were precipitated with NeutrAvidin beads and subjected to immunoblotting analysis. CAPE (1 μM) was added to cell lysates treated with BT-CA. (**E**) Sensograms of CAPE binding to recombinant ASC protein in the presence of detergent (0.005% Tween-20) were obtained from surface plasmon resonance (SPR) analysis. Different concentrations of CAPE are presented as an overlay plot aligned at the start of injection. (**F**) The line graph of dose-binding response unit curve and the table showing kinetic parameters of the binding between CAPE and ASC calculated using a simple 1:1 interaction model were from SPR analysis in (**E**). The maximal expected binding level (Rmax^c^) was calculated by Biocore T200 evaluation software and Rmax^e^ value was obtained from experimental maximum response unit. Veh, vehicle. P, precipitation. IB, immunoblotting. WB, western blotting.

To investigate whether CAPE binds to ASC, we performed pulldown experiments using biotin-tagged caffeic acid (BT-CA) (Fig. 5A). BT-CA exerted the inhibitory activity for MSU crystals-induced IL-1β secretion in BMDMs (Fig. 5B). We generated the structural analogs of BT-CA to examine the relationship between the inhibitory effect on IL-1β production and the binding activity to ASC. Biotin- tagged dihydrodihydroxycinnamic acid phenethyl ester (BT-DHC) inhibited IL-1β production induced by MSU crystals, while biotin-tagged dimethoxycinnamic acid phenethyl ester (BT-DMC) did not show such activity (Fig. 5A and B). To examine whether BT-CA bound to ASC in the cell, BMDM cell lysates were treated with BT-CA. BT-CA bound proteins were precipitated with NeutrAvidin-beads and subjected to immunoblotting analysis for ASC. ASC was detected in the precipitated proteins, showing that BT-CA bound to ASC (Fig. 5C), suggesting that CAPE binds to ASC in BMDMs. Addition of CAPE attenuated precipitation of ASC with BT-CA, showing that CAPE prevented the binding of BT-CA to ASC (Fig. 5C). ASC was detected in NeutrAvidin-precipitated proteins derived from BMDM lysates incubated with BT-DHC, but not BT-DMC (Fig. 5C). These suggest that the inhibitory effects of the CAPE analogs on IL-1β production are correlated with their binding capacity to ASC.

To further confirm the binding of CAPE to ASC, BT-CA was treated with 293T cell lysates exogenously expressing ASC by transfection with ASC-expression plasmid. Immunoblotting analysis with NeutrAvidin-beads-precipitated proteins showed that BT-CA bound to ASC in 293T cell lysates expressing exogenous ASC (Fig. 5D). Consistently with the results with Fig. 5C, addition of CAPE abolished the precipitation of ASC with BT-CA in 293T cell lysates (Fig. 5D). Exogenously expressed ASC was co-precipitated with BT-DHC, but not BT-DMC (Fig. 5D). We investigated whether CAPE abolished ASC speck formation in 293T cells overexpressing ASC. After 293T cells were transfected with ASC expression plasmid and treated with CAPE, ASC speck formation was examined by confocal microscopy analysis. CAPE treatment resulted in decrease of ASC speck formation in 293T cells overexpressing ASC (Supplemental Figure 7).

These results show that BT-CA associates with both endogenously and exogenously expressed ASC, suggesting that CAPE targets and binds to ASC in the cell.

To further confirm the binding of CAPE to ASC, we employed SPR analysis with recombinant ASC protein. CAPE directly bound to ASC in a dose-dependent manner (Fig. 5E and F). The parameters of the interaction kinetics and the affinity constants were calculated based on a simple 1:1 interaction model using Biacore T200 software and are presented in Fig. 5F.

We investigated whether CAPE affected activation of other inflammasomes that require ASC. AIM2 (absent in melanoma 2), an interferon-inducible HIN-200 family member, senses cytoplasmic double-stranded DNA, forming an inflammasome with ASC via homotypic PYD-PYD interactions to induce the activation of caspase-1^14^. We examined whether CAPE regulated activation of AIM2 inflammasome induced by transfection of synthetic double-stranded DNA, poly dA:dT in LPS-primed BMDMs. CAPE decreased the degradation of pro-caspase-1 to caspase-1(p10) and cleavage of pro-IL-1β to IL-1β induced by poly dA:dT in BMDMs as demonstrated by immunoblotting and ELISA (Supplemental Figure 8). These results demonstrate that CAPE suppressed the activation of other inflammasomes such as AIM2 inflammasome, which require ASC.

### CAPE blocks the interaction between NLRP3 and ASC

**Figure 6:**
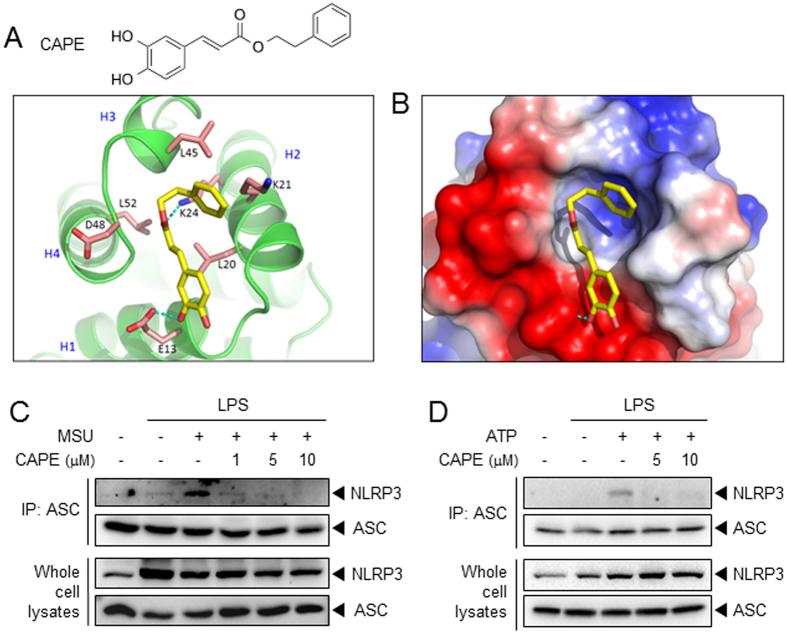
CAPE blocks the interaction between NLRP3 and ASC. (**A**) The chemical structure of CAPE and the proposed molecular docking model for CAPE binding to ASC. (**B**) Electrostatic surface binding model for CAPE and ASC. Red: negative charge, blue: positive charge. (**C,D**) BMDMs were primed with LPS (**C**, 500 ng/ml; **D**, 100 ng/ml) for 4 hr. Then, the cells were treated with CAPE for 1 hr, followed by stimulation with MSU (500 μg/ml) for 5 hr or ATP (5 mM) for 1 hr. Cell lysates were immunoprepitated with anti-ASC antibody followed by immunoblotting as indicated. Representative data from at least two independent experiments are presented.

To investigate the ASC domain to which CAPE binds, we performed SPR analysis using recombinant proteins of ASC-PYD, ASC-CARD, and NLRP3-PYD. CAPE bound to ASC-PYD similarly with ASC protein (Supplemental Figure 9A). However, CAPE did not bind to ASC-CARD or NLRP3-PYD (Supplemental Figures 9B and 9C). The results suggest that CAPE preferentially binds to ASC-PYD. Molecular modeling analysis using the crystal structure of ASC (2KN6), which was obtained from the protein data bank (PDB)^15^, suggests a docking model between CAPE and the PYD domain of ASC. CAPE formed hydrogen bonds with Glu13 and Lys24 of ASC and interacted with Lys21 and Leu45 through a lipophilic interaction (Fig. 6A and B). In particular, Glu13 is an important amino acid that plays a critical role in both NLRP3-ASC interaction^16^ and ASC-ASC oligomerization^17^.

Therefore, we asked whether the binding of CAPE to ASC would result in the disruption of the NLRP3-ASC interaction. Co-immunoprecipitation study showed that CAPE prevented MSU-induced association of NLRP3 and ASC in BMDMs (Fig. 6C). In addition, ATP-induced association between NLRP3 and ASC was also blocked by CAPE (Fig. 6D). These results suggest that CAPE suppresses the activation of the NLRP3 inflammasome by directly binding ASC, blocking the association of NLRP3 and ASC.

Together, oral administration of CAPE effectively attenuated the inflammatory symptoms of gouty arthritis by suppressing NLRP3 inflammasome activation. CAPE targeted ASC, a bridge protein for NLRP3 and caspase-1, thereby blocking NLRP3 inflammasome activation.

## Discussion

In this study, we presented that CAPE would be effective to prevent acute gout by targeting ASC and inhibiting NLRP3 inflammasome activation, as CAPE administration reduced inflammatory symptoms in two animal models of acute gout. Both *in vitro* studies on primary macrophages and *in vivo* studies using animal models, including an air pouch model -which mimics the synovium-and a foot gout model showed that CAPE’s inhibitory effects were mediated by the suppression of uric acid crystal-induced NLRP3 inflammasome activation. In addition, CAPE’s suppression of NLRP3 inflammasome activation was supported *in vivo* by luminescence imaging analysis using the iGLuc luciferase reporter. CAPE’s applications could be extended to the treatment of other diseases related to uric acid crystal accumulation. It has been reported that uric acid released from injured cells contributes to lung injury-associated inflammation and fibrosis via activation of the NLRP3 inflammasome^18^. In future study, it would be worth examining the efficacy of CAPE treatment against uric acid-mediated lung diseases.

Recent studies have reported that certain phytochemicals, such as resveratrol^19^, quercetin^20^ and epigallocatechin-3-gallate^21^, can inhibit NLRP3 inflammasome activation by blocking mitogen-activated protein kinase (MAPK) and nuclear factor (NF)-κB activation or by decreasing reactive oxygen species (ROS) production. Other small-molecule inhibitors, such as 3,4-methylenedioxy-β-nitrostyrene^22^, MCC950^23^, and β-hydroxybutyrate^24^, have also been reported to suppress NLRP3 inflammasome activation. In this study, we propose ASC as a new regulatory target of anti-inflammatory phytochemical, CAPE, in NLRP3 inflammasome pathway. Our overexpression study indicated ASC as a required component for CAPE’s inhibitory effect on NLRP3 inflammasome. CAPE did not directly bind to NLRP3 PYD, nor inhibited caspase-1 enzyme activity. These suggest that CAPE preferentially targets ASC among NLRP3 inflammasome components. Although several previous studies have investigated inhibitory chemicals, to the best of our knowledge, this report is the first showing the direct association of an anti-inflammatory phytochemical with ASC resulting in the inhibition of the NLRP3 inflammasome.

It is well known that CAPE suppresses the activation of transcription factors, thereby regulating the transcription levels of cytokines. However, in this study, we intended to elucidate the direct effect of CAPE on inflammasome activation rather than the effect on the transcriptional levels of pro-caspase-1 and pro-IL-1β expression. Therefore, CAPE was treated after washing out LPS in BMDMs to dissect CAPE’s effect on the transcription of pro-IL-1β. The cleavage of pro-caspase-1 and pro-IL-1β to caspase-1(p10) and IL-1β are the hallmarks of inflammasome activation and that these cleavages are blocked by CAPE. These suggest the direct regulatory role of CAPE in inflammasome activation, which was clearly demonstrated by immunoblotting experiments with BMDM cells and *in vivo* luminescence-imaging study using an inflammasome-dependent reporter plasmid. Finally, our results indicate the direct binding of CAPE to ASC-PYD, leading to the disruption of NLRP3-ASC association. It is still possible that CAPE would exert both the transcriptional regulation and the direct impact on inflammasome component at *in vivo* situation where the priming signal and the inflammasome activating signal are mixed.

Collectively, our results show that CAPE, a natural product that is abundant in propolis, is a small-molecule inhibitor of the NLRP3 inflammasome. Thus, CAPE may have preventive or therapeutic potential against NLRP3 inflammasome-related diseases, particularly gout. CAPE directly associates with ASC, thereby blocking the assembly of NLRP3-ASC. Thus, ASC could be a new therapeutic target for gout. Our results reveal a new regulatory mechanism that modulates the activation of the NLRP3 inflammasome and can be utilized as the basis for development of new NLRP3 inflammasome inhibitors.

## Methods

### Ethics statement

All animals received humane care according to the criteria outlined in the “Guide for the Care and Use of Laboratory Animals” prepared by the National Academy of Sciences and published by the National Institutes of Health (NIH publication 86–23 revised 1985). All the experimental procedures were carried out in accordance with the protocols approved by the Institutional Animal Care and Use Committee (IACUC) of the Catholic University of Korea (permission# 2014–015).

### Animals and cell culture

Mice(C57BL/6) were obtained from Orient Bio (Seoul, Korea). Nlrp3^A350VneoR^ mice (B6.129-*Nlrp3*^*tm1Hhf*^/J, Stock Number:017969) were purchased from Jackson Laboratory (Bar Harbor, ME). The mice were housed in a room controlled for temperature (23 ± 3 °C) and relative humidity (40–60%) under specific pathogen-free conditions. Mice were acclimated in the animal facility for at least a week before the experiments. Mice of individual experimental groups in each experiment were of similar age and weight and randomly allocated to treatment groups. Investigators were blinded for treatment or genotype of mice, or both, in all experiments. Bone marrow-derived primary macrophages (BMDMs) were prepared from mice as described previously^25^. Bone marrow-derived immortalized macrophages from C57BL/6j mice were kindly provided by S. Kim (The Western University, Canada)^26^. Macrophages and 293T cells (human embryonic kidney cells) were cultured in Dulbecco’s modified eagle medium containing 10%(v/v) fetal bovine serum (Invitrogen, Carlsbad, CA), 10,000 units/ml of penicillin, and 10,000 μg/ml of streptomycin.

### Reagents

Purified LPS from *Escherichia coli* was obtained from List Biological Laboratory Inc. (Campbell, CA). CAPE was purchased from Sigma-Aldrich. The structural derivatives of CAPE, biotin tagged CA, -DHC, and -DMC were synthesized as described previously^27^. Monosodium urate (MSU) and ATP were purchased from Invivogen (Carlsbad, CA). Antibodies for mouse caspase-1 and ASC were obtained from Santa Cruz Biotechnology (Santa Cruz, CA). Antibody for NLRP3 was purchased from Adipogen (San Diego, CA). Antibody for IL-1β was from R&D Systems (Minneapolis, MN). Caspase-1 activity kit for animal model was from Ab-cam (Cambridge, MA).

### Plasmids

A pcDNA3.1nV5-hNLPR3 expression plasmid was from You-Me Kim (Pohang University of Science and Technology, South Korea). Expression plasmids for ASC and caspase-1 were gifts from Giulio Superti-Furga (Austrian Academy of Sciences, Austria). An iGLuc plasmid was provided by Veit Hornung (University of Bonn, Germany). Transient transfection and luciferase assay were performed as previously described^25^.

### Analysis of inflammasome activation

This was performed as previously described^28^. Briefly, BMDMs were primed with LPS for 4 hrs. To exclude the effect of CAPE on LPS, CAPE was added after washing out the LPS with phosphate-buffered saline(PBS). The cells were treated with CAPE and stimulated with NLRP3 inflammasome activators such as MSU and ATP in serum-free medium. The cells were lysed in RIPA buffer (50 mM Tris-HCl, pH 7.4, 1% NP-40, 0.25% sodium deoxycholate, 150 mM NaCl, 1 mM EGTA, 1 mM PMSF, 1 mM Na_3_VO_4_, 10 μg/ml aprotinin, 10 μg/ml leupeptin). The supernatants were precipitated with methanol: chloroform (1:0.25), followed by centrifugation at 20,000 g for 10 min. The upper phase was discarded and one volume of methanol was added. The mixture was centrifuged at 20,000 g for 10 min to obtain a protein pellet, which was dried at room temperature and resuspended in Laemmli buffer (0.25 M Tris-HCl, pH 6.8, 0.4% glycerol, 10% SDS, 0.2% 2-mercaptoethanol, 0.64% bromophenol blue). The samples were resolved with SDS-PAGE and subjected to immunobloting assay.

### Enzyme-linked immunosorbent assays

Levels of IL-1β, IL-18 and TNF-α were determined using enzyme-linked immunosorbent assay (ELISA) kits (R&D systems, Minneapolis, MN), according to the manufacture’s instruction. The levels of IL-1α, IL-6, KC and MCP-1 were measured using a MILLIPLEX MAP Mouse Cytokine/Chemokine Kit (Millipore, Billerica, MA). The fluorescent intensity was read on a MAGPIX® (Luminex Corporation, Austin, TX).

### Air pouch inflammation model

The backs of mice (7 to 8 weeks old) were subcutaneously injected with 5 ml of sterile air to make an air pouch, and an additional 5 ml of sterile air was injected into the pouch 3 days later. On day 4, 0.2 ml sterilized water containing the vehicle or CAPE was orally administered, and MSU crystals were injected 1 hr later. Six hours after the injection of the MSU crystals, the mice were anesthetized, and the air pouch fluids were lavaged with 2 ml PBS containing 5 mM EDTA. The lavages were centrifuged at 1200 rpm for 2 min, and the supernatants were used for ELISAs. For immunoblot assays, air pouch lavages were precipitated to obtain protein pellets. For histologic analysis, sagittal sections of air pouches were fixed in 10% paraformaldehyde and stained with hematoxylin and eosin (H&E).

### Luminescence-based *in vivo* imaging analysis

Bone marrow-derived immortalized macrophages were transiently transfected with iGLuc plasmid as described previously^13^. After the cells were injected into air pouches on the backs of mice, mice were orally administered with CAPE. One hour later, the mice were injected with either 1 ml of PBS alone or PBS containing MSU crystals. After 6 hr, mice were injected with 1 ml of luciferase substrate (Renilla-Glo® Luciferase Assay system, Promega, cat.#E2720, Madison, WI) and subjected to luminescence measurements using an Xtreme system (Bruker, Billerica, MA). iGLuc signal was measured over 5 min of exposure with the acquisition mode set to luminescence and photography overlay.

### Myeloperoxidase (MPO) activity assay

MPO activity was determined using MPO colorimetric activity assay kit (Bio-vision, Milpitas, CA).

### A foot gout model in mice

Mice (7 to 8 weeks old) were orally administered 0.5 ml sterilized water containing CAPE or vehicle. After 1 hr, MSU crystals or PBS were subcutaneously injected under the plantar surface of the right paw. Twenty-four hours after injecting MSU crystals, foot tissue was homogenized in RIPA buffer, and the supernatant was collected for MPO assays, ELISAs and immunoblot assays. For histological analysis, sagittal sections of the footpads were fixed in 10% paraformaldehyde and stained with H&E.

### Immunoblots of ASC monomer and oligomer

Insoluble ASC complexes were isolated from cell lysates by centrifugation and subsequent crosslinking with discuccinimidyl suberate (Thermo Scientific, Waltham, MA) as previously described^10^. The samples were resolved on SDS-PAGE and processed for immunoblot assay.

### Confocal microscopy analysis

Confocal microscopy analysis was performed as previously described^29^. Briefly, BMDMs were plated on coverslips and incubated with an anti-ASC antibody, then incubated with an anti-rabbit IgG-FITC antibody. The cells were examined with an LSM710 laser scanning confocal microscope (Carl Zeiss, Oberkochen, Germany) using Zen2010 software.

### Surface plasmon resonance (SPR) study

Anti-ASC antibodies were covalently immobilized to a CM5 sensor chip (cat.#BR-1005-30, GE Healthcare, Buckinghamshire, UK). Recombinant human ASC protein(Abnova) was captured with anti-ASC antibodies via antibody-antigen binding. For affinity measurements, the association and dissociation phases were monitored with a Biacore T200 (GE Healthcare). CAPE was dissolved in basic running buffer (PBS containing 0.005% Tween-20 and 5% DMSO) and injected into the flow cell at different concentrations with a flow rate of 5 μl/min at 25 °C. The sensor chip was washed with basic running buffer between each concentration. Control experiments were performed with blank (sensor chip only) and active(sensor chip with antibody only) channels on the same sensor chip. Based on the obtained assay curves, the control signals, which reflected the bulk effect of the buffer, were subtracted using T200 evaluation software ver. 2.0 (GE Healthcare). The kinetic parameters of the interaction and the affinity constants were calculated using a simple 1:1 interaction model with Biacore T200 evaluation software.

### Molecular modeling study

To predict a model of CAPE binding to ASC, we used the crystal structure of ASC (PDB code 2KN6). The protein structure was minimized using the Protein Preparation Wizard in the Maestro graphical user interface (version 9.3, Schrödinger). CAPE was docked into a minimized crystal structure of the PYD of the ASC using the docking routine in Prime (ver.3.1, Schrödinger, LLC, New York, NY, 2012). The docking was performed using the default settings and keeping all residues fixed. The graphics for the refined docking model for CAPE were generated using PyMol (http://www.pymol.org).

### Precipitation and immunoblotting of proteins associated with biotin-tagged compounds

Cell lysates were treated with biotin-tagged CA, -DHC, or -DMC at room temperature for 4 hr and further incubated with NeurAvidin-beads (Thermo Scientific) at 4 °C for 2 hr. The samples were centrifuged at 15,000 g for 5 min and washed with TENT buffer (50 mM Tris, pH 7.0, 5 mM EDTA, 150 mM NaCl, 0.05% Tween 20) three times. Proteins co-precipitated with the beads were processed for immunoblot assays.

### Statistical analysis

Statistical analysis was performed using the software GraphPad Prism 7(GraphPad Software, San Diego, CA). All data were expressed as means ± SEM and analyzed for normality by Kolmogorov–Smirnov test before applying parametric statistical tests. Datasets were analyzed by one-way ANOVA (followed by Turkey’s multiple comparison test), after being tested for differences in their variance by Brown-Forsythe test to ensure that groups of data with unequal sample sizes had similar variance. P-values < 0.05 were considered significant.

## Additional Information

**How to cite this article**: Lee, H. E. *et al*. Targeting ASC in NLRP3 inflammasome by caffeic acid phenethyl ester: a novel strategy to treat acute gout. *Sci. Rep.* **6**, 38622; doi: 10.1038/srep38622 (2016).

**Publisher's note:** Springer Nature remains neutral with regard to jurisdictional claims in published maps and institutional affiliations.

## Supplementary Material

Supplementary Materials
