# Supplementary material for: Targeting ASC in NLRP3 inflammasome by caffeic acid phenethyl ester: a novel strategy to treat acute gout
Source: Sci Rep. 2016 Dec 9;6:38622. doi: 10.1038/srep38622 (PMC5146947; doi:10.1038/srep38622)
Supplement: Supplementary Materials [file srep38622-s1.doc]

**Supplementary materials**

**Targeting ASC in NLRP3 inflammasome by caffeic acid phenethyl ester: a novel strategy to treat acute gout**

Hye Eun Lee^1‡^, Gabsik Yang^1‡^, Nam Doo Kim^2^, Seongkeun Jeong^3^, Yunjin Jung^3^, Jae Young Choi^4^, Hyun Ho Park^4^, and Joo Young Lee^1*^

^1^BK21plus team, College of Pharmacy, The Catholic University of Korea, Bucheon, Republic of Korea, 14662

^2^Daegu-Gyeongbuk Medical Innovation Foundation, New Drug Development Center, Daegu, Korea, 41061

^3^College of Pharmacy, Pusan National University, Busan, Republic of Korea 46241

^4^School of Chemistry and biochemistry and Graduate school of Biochemistry, Yeungnam University, Gyeongsan, Republic of Korea 38541

^*^Corresponding author: Joo Young Lee, College of Pharmacy, The Catholic University of Korea, Bucheon, Korea, 14662.

Tel: 82-2-2164-4095. Fax: 82-2-2164-4059. E-mail: joolee@catholic.ac.kr.

^‡^Hye Eun Lee and Gabsik Yang equally contributed.

**
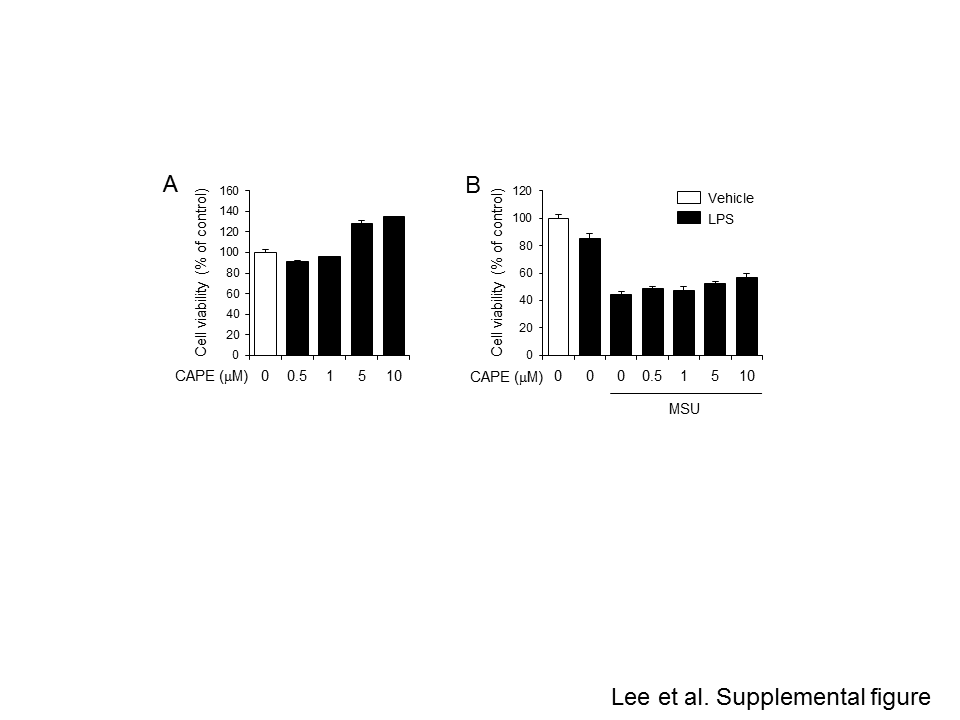
**

**Supplemental Figure 1. CAPE does not affect cell viability.** Bone marrow-derived macrophages were treated with (A) CAPE alone or (B) CAPE in the presence of LPS and/or MSU for 12 hr. Cell viability was determined by MTT assay. The values are expressed as the % of control (vehicle). The values represent the means ± SEM (n=6).

**
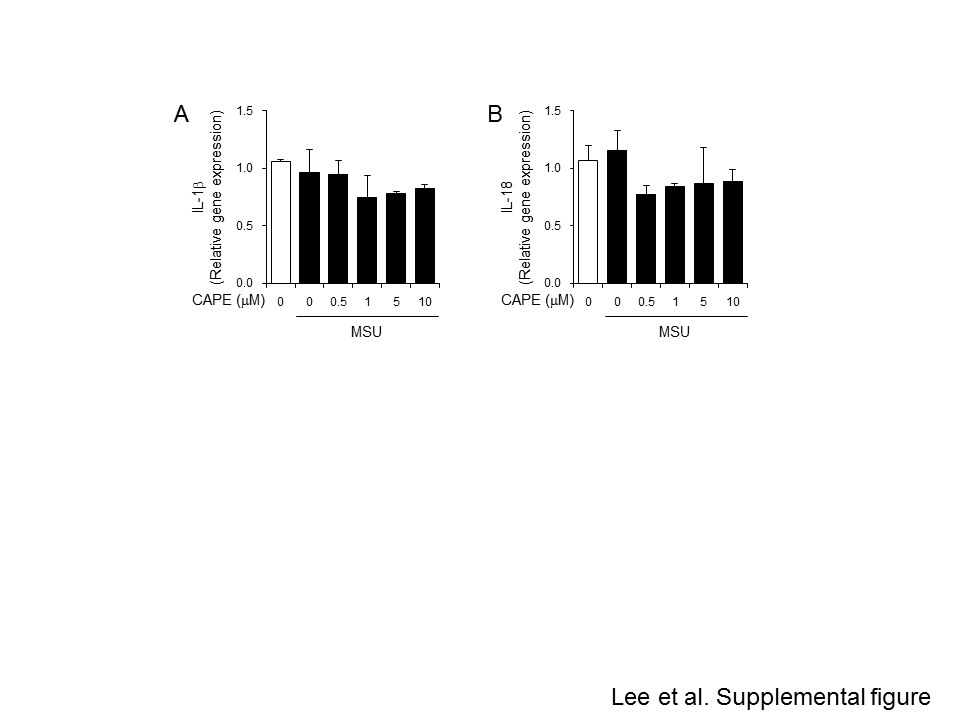
**

**Supplemental Figure 2. CAPE did not affect the mRNA levels of IL-1β and IL-18 in bone marrow-derived macrophages stimulated with MSU.** Bone marrow-derived macrophages were primed with LPS (500 ng/ml) for 4 hr. After washing the cells, cells were treated with CAPE for 1 hr and then stimulated with monosodium uric acid (MSU) crystals (500 μg/ml) for 6 hr. The mRNA levels of IL-1β and IL-18 were determined by quantitative real time PCR analysis and are expressed as the relative expression levels compared to vehicle for each gene. The values represent the means ± SEM (n=3).

**
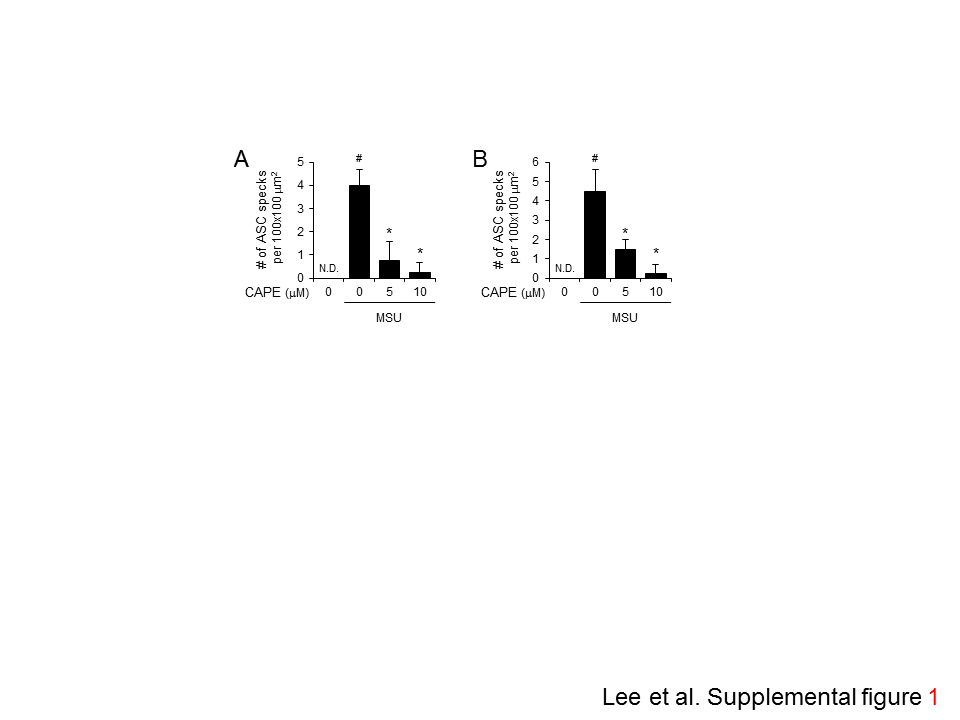
**

**Supplemental Figure 3. Quantification of the number of ASC specks.** The confocal microscopy analysis was performed as described in Figure 1F. The number of ASC specks per 100x100 μm^2^ was obtained from (A) different fields of view and (B) a different preparation. The values represent the means ± SEM (n=3). #, significantly different from vehicle alone, p <0.05. *, significantly different from MSU alone, p <0.05. N.D. not detected.


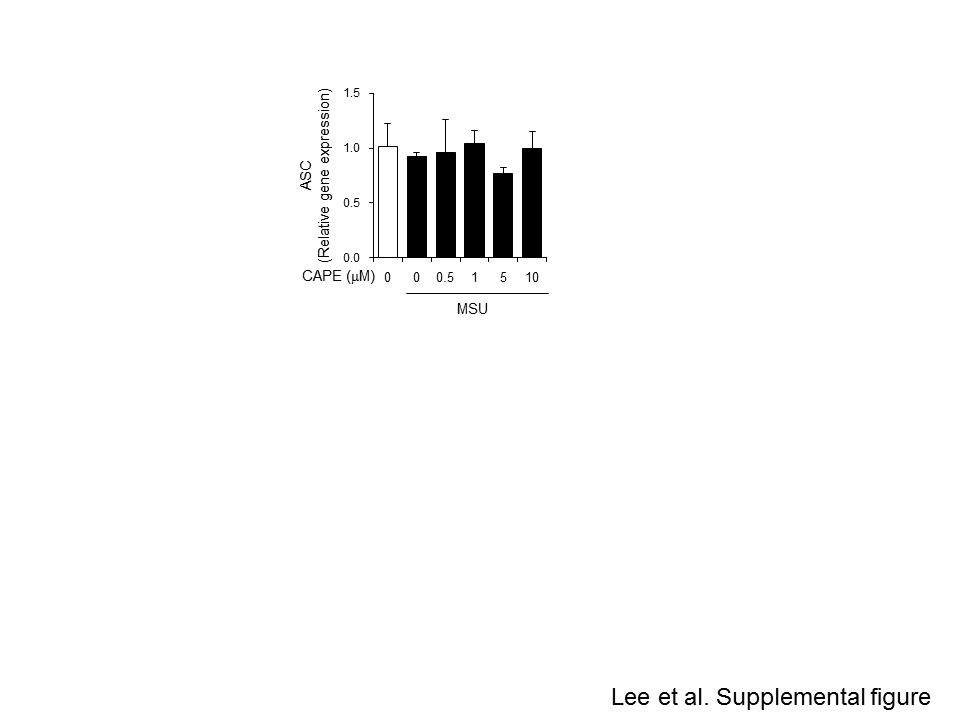


**Supplemental Figure 4. CAPE did not affect the mRNA levels of ASC in bone marrow-derived macrophages stimulated with MSU.** The cells were treated as described in Supplemental Figure 2. The mRNA levels of ASC were determined by quantitative real time PCR analysis and are expressed as the relative expression levels compared to vehicle for each gene. The values represent the means ± SEM (n=3).

**
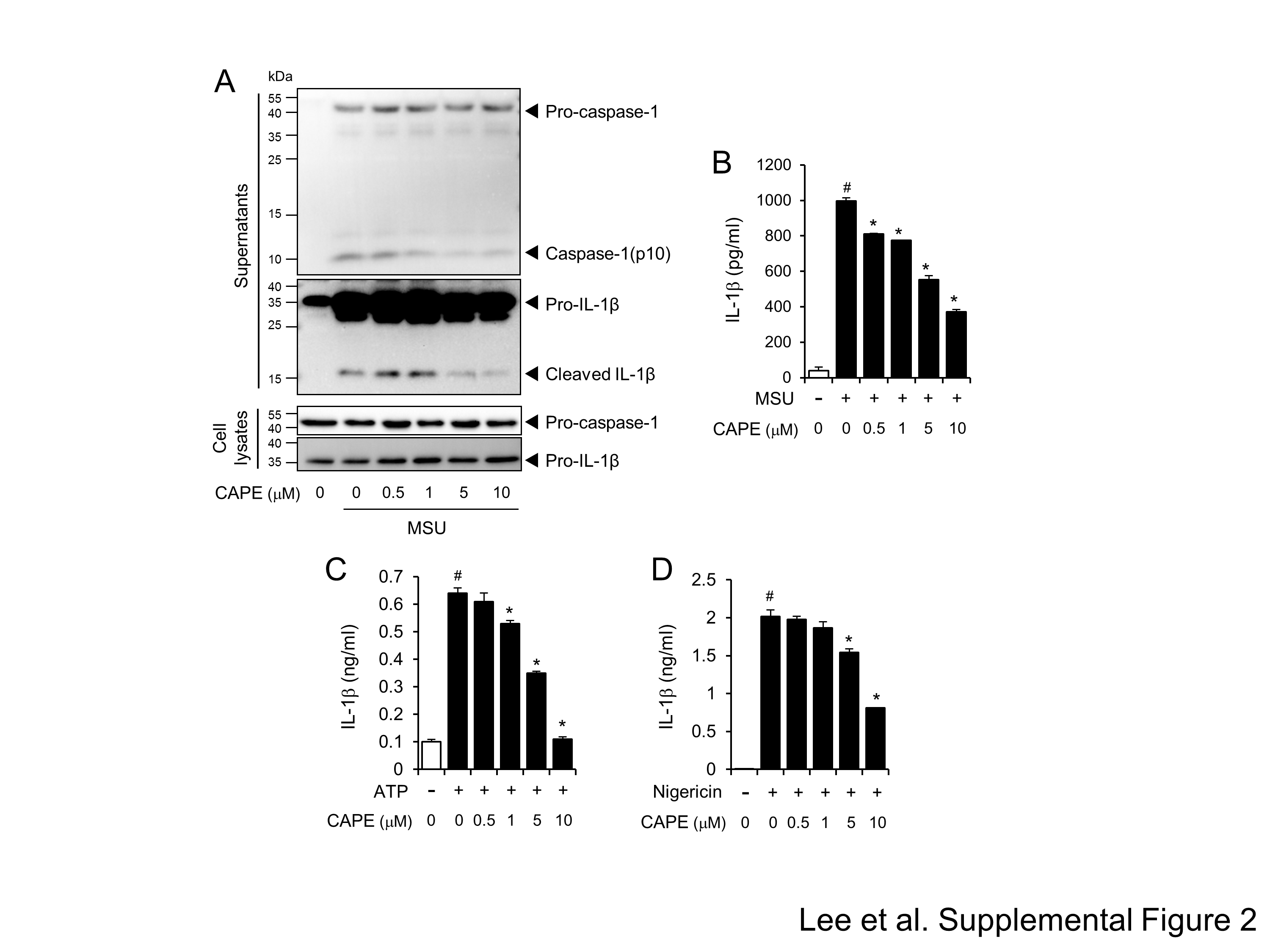
**

**Supplemental Figure 5. CAPE suppresses activation of the NLRP3 inflammasome in human monocytic cells.** THP-1 cells (human monocytic cell line) were cultured in RPMI1640 medium (Invitrogen) containing 50 μM β-mercaptoethanol (Sigma-Aldrich, St. Louis, MO), 10 % (v/v) fetal bovine serum, 10,000 units/ml of penicillin, and 10,000 μg/ml of streptomycin. **A.** The cell culture supernatants and cell lysates were immunoblotted for pro-caspase-1, caspase-1(p10), pro-IL-1β, and IL-1β. **B.** Cell culture supernatants were analyzed for secreted IL-1β by ELISA. The values represent the means ± SEM (n=3). #, significantly different from vehicle alone, p <0.05. *, significantly different from MSU alone, p <0.05.

**
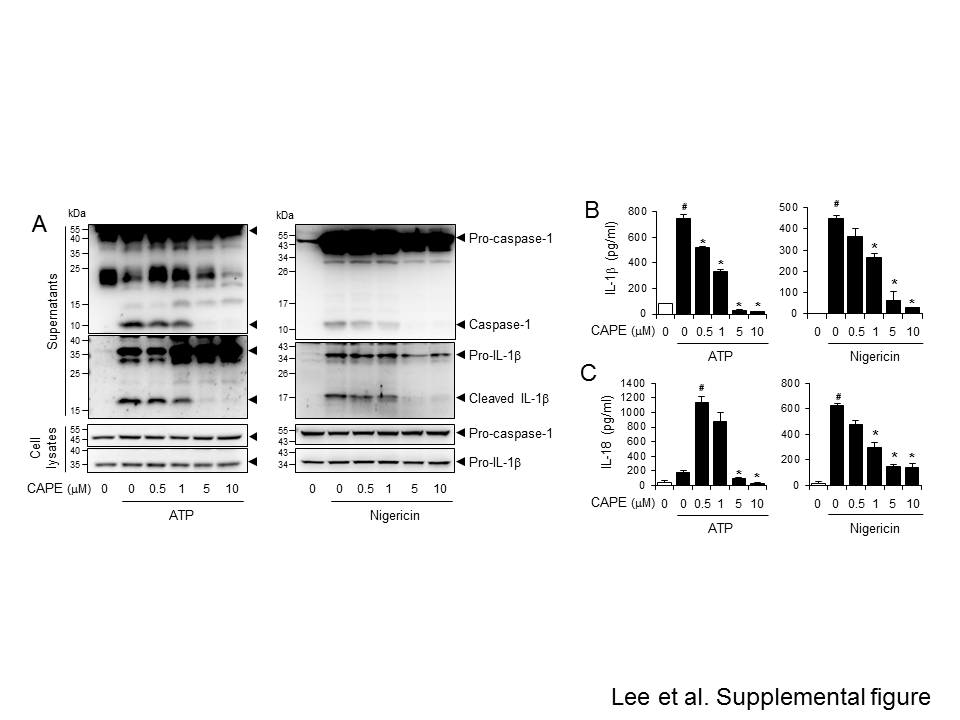
**

**Supplemental Figure 6. CAPE suppresses ATP- and nigericin-induced activation of the NLRP3 inflammasome in primary macrophages.** Bone marrow-derived macrophages were primed with LPS (100 ng/ml) for 4 hr. Then, the cells were treated with CAPE for 1 hr, followed by stimulation with ATP (5 mM) or nigericin (10 μM). In A, the cell culture supernatants and cell lysates were collected 1 hr after ATP or nigericin and immunoblotted for pro-caspase-1, caspase-1(p10), pro-IL-1β, and IL-1β. In B, the cell culture supernatants were collected 2 and 16 hr after ATP and nigericin treatment, respectively, and analyzed for secreted IL-1β using ELISA. In C, the cell culture supernatants were collected 1 and 2 hr after ATP and nigericin treatment, respectively, and analyzed for secreted IL-18 using ELISA. The values represent the means ± SEM (n=3). #, significantly different from vehicle alone, p <0.05. *, significantly different from ATP or nigericin alone, p <0.05.


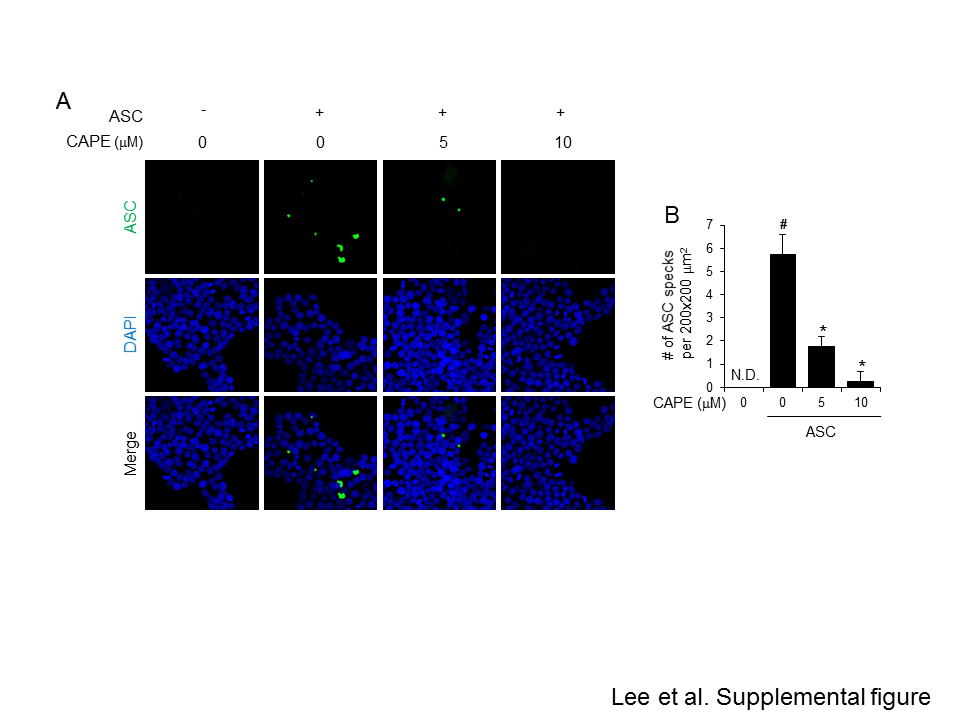


**Supplemental Figure 7. CAPE decreases ASC speck formation in 293T cells overexpressing ASC.** After 293T cells were transfected with an expression plasmid of ASC for 9 hr, cells were treated with CAPE for 16 hr. The cells were fixed, permeabilized and stained for ASC (green) and the nuclei were stained with 4',6-diamidino-2-phenylindole (DAPI; blue). (A) The representative images were presented. (B) The number of ASC specks was counted from three different fields of view in 200x200 μm^2^. The values represent the means ± SEM (n=3). #, significantly different from vehicle alone, p <0.05. *, significantly different from ASC alone, p <0.05. N.D. not detected.


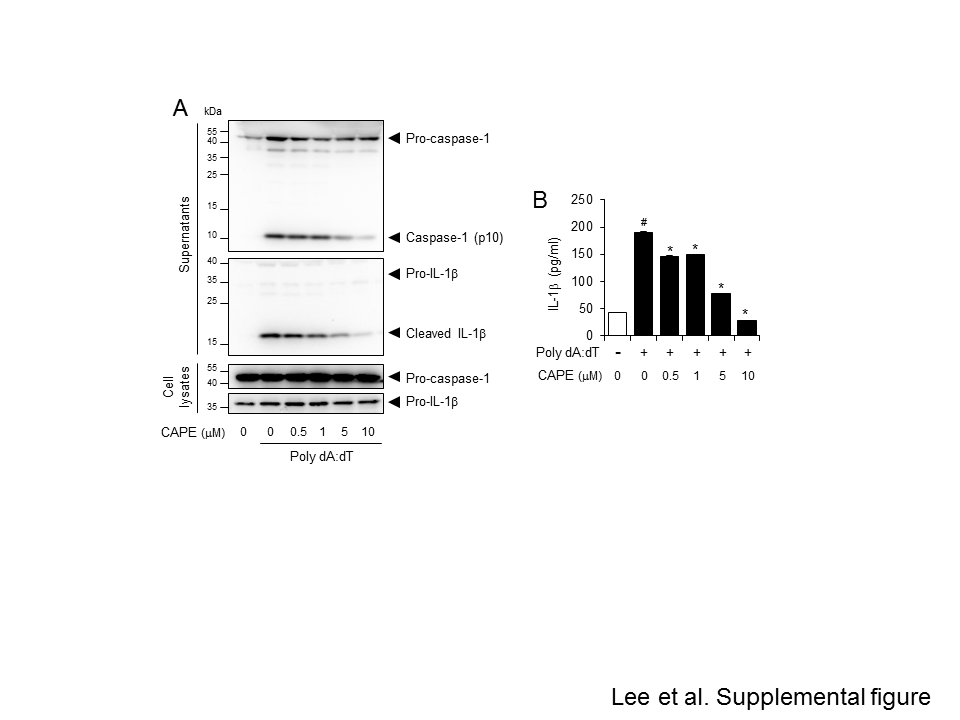


**Supplemental Figure 8. CAPE suppresses activation of AIM2 inflammasome in primary macrophages.** Bone marrow-derived macrophages were primed with LPS (500 ng/ml) for 4 hr. Then, the cells were treated with CAPE for 1 hr and further stimulated with transfection of poly dA:dT (1 μg). **A.** Cell culture supernatants and cell lysates were collected 4.5 hr after poly dA:dT, and immunoblotted for pro-caspase-1, caspase-1(p10), pro-IL-1β, and IL-1β. **B.** Cell culture supernatants were collected 6 hr after poly dA:dT transfection, and analyzed for secreted IL-1β using ELISA. The values represent the means ± SEM (n=3). #, significantly different from vehicle alone, p <0.05. *, significantly different from poly dA:dT alone, p <0.05.


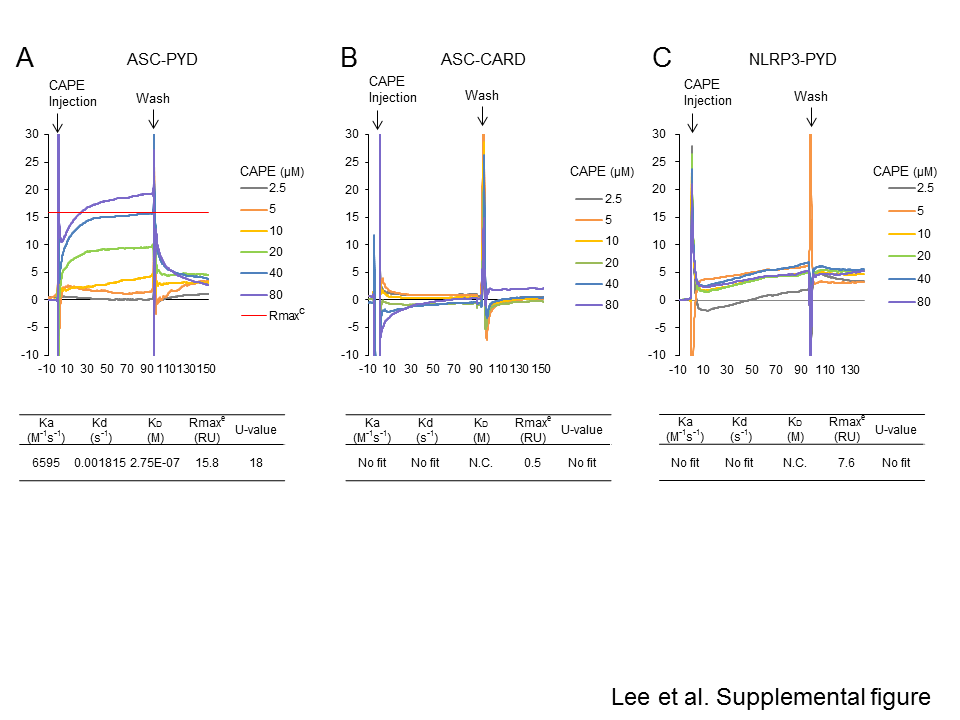


**Supplemental Figure 9. CAPE binds to ASC-PYD, but not ASC-CARD nor NLRP3-PYD.** Sensograms of CAPE binding to each recombinant protein, (A) ASC-PYD, (B) ASC-CARD, and (C) NLRP3-PYD in the presence of detergent (0.005% Tween-20) were obtained by surface plasmon resonance (SPR) analysis. Different concentrations of CAPE are presented as an overlay plot aligned at the start of injection. Table of kinetic parameters were obtained from SPR analysis of CAPE interaction to each recombinant protein. The maximal expected binding level (Rmax^c^) was calculated by Biocore T200 evaluation software.
